# Synthesis of Taxifolin-Loaded Polydopamine for Chemo-Photothermal-Synergistic Therapy of Ovarian Cancer

**DOI:** 10.3390/molecules29051042

**Published:** 2024-02-28

**Authors:** Yang Lu, Xinglong Liu, Ting Zhao, Chuanbo Ding, Qiteng Ding, Ning Wang, Shuang Ma, Lina Ma, Wencong Liu

**Affiliations:** 1 School of Resources and Environment, Jilin Agricultural University, Changchun 130118, China; luyang01023@163.com (Y.L.);; 2 School of Traditional Chinese Medicine, Jilin Agriculture Science and Technology College, Jilin 132101, China; 3 School of Food and Pharmaceutical Engineering, Wuzhou University, Wuzhou 543002, China

**Keywords:** PDA, photothermal agent nanoparticles, TAX, ovarian cancer

## Abstract

Chemotherapy is a well-established method for treating cancer, but it has limited effectiveness due to its high dosage and harmful side effects. To address this issue, researchers have explored the use of photothermal agent nanoparticles as carriers for precise drug release in vivo. In this study, three different sizes of polydopamine nanoparticles (PDA–1, PDA–2, and PDA–3) were synthesized and evaluated. PDA–2 was selected for its optimal size, encapsulation rate, and drug loading rate. The release of the drug from PDA–2@TAX was tested at different pH and NIR laser irradiation levels. The results showed that PDA–2@TAX released more readily in an acidic environment and exhibited a high photothermal conversion efficiency when exposed to an 808 nm laser. In vitro experiments on ovarian cancer cells demonstrated that PDA–2@TAX effectively inhibited cell proliferation, highlighting its potential for synergistic chemotherapy-photothermal treatment.

## 1. Introduction

Ovarian cancer (OC) is considered the most lethal gynecologic malignancy due to its late detection, high risk of recurrence, and resistance to modern chemotherapeutic drugs [1,2,3]. It ranks as the third most common malignancy of the female reproductive system, following uterine corpus and cervical malignancies. Unfortunately, OC has one of the lowest survival rates among gynecological malignancies, primarily because of its aggressive nature and the difficulties associated with its identification. Chemotherapy [4], radiation [5], surgery [6], and combination therapies [7] are frequently used to treat OC. Traditional chemotherapy operations for tumor treatment often lead to the development of drug resistance in cancer cells, which can result in cancer recurrence as the resistant cells continue to grow [8]. Therefore, it is crucial to promptly identify innovative treatments for ovarian cancer. Recently, there has been significant interest in the development of tumor-targeted and controlled-release medication nano delivery devices for cancer treatment [9]. As currently known, micro multifunctional platforms that are sensitive to internal or external stimuli [10], and photothermal [11], acid-base [12], and redox conditions [13] can provide exact releases to the location.

Near-infrared responsive (NIR) photodynamic therapy has been shown to be effective in treating deep tissues [14]. Upon illumination of the target region, the photothermal agent converts light energy into heat, leading to the destruction of cancer cells. Clinical studies have indicated that these delivery methods are more effective and have fewer harmful side effects, compared to traditional chemotherapy [11,15]. Examples of therapies used to treat cancer include photodynamic therapy (PDT) [16], photothermal therapy (PTT) [17], chemodynamic therapy (CDT) [18], gene therapy [19], etc. According to several studies, various nanomaterials have been found to have a warming and drug release-promoting effect when illuminated, and have the ability to kill cancer cells at temperatures as high as 45 °C [20,21,22]. These techniques, known as anticancer photothermal therapy, utilize laser irradiation to destroy cancer cells. This approach has gained significance as a synergistic therapy due to its advantages, such as minimal systemic harm and limited adverse effects.

Polydopamine (PDA) is a well-known photothermal material that has garnered significant interest due to its easy synthesis, excellent photostability, good biodegradability, and remarkable biocompatibility properties [23,24]. It is highly sought in the field of photothermal materials because of its simple synthesis, superior photostability, good biodegradability, and excellent biocompatibility attributes. This is attributed to the presence of amines, imines, catechols, and other substances in polydopamine, which allow it to exhibit a strong adhesion capability and the ability to be adsorbed on the surface of any solid substance [25,26,27].

Taxifolin (TAX) is a flavonoid with a unique structure that exhibits greater biological activity and antioxidant capacity, compared to other flavonoids [28]. Over the years, numerous studies have demonstrated the anticancer, antiviral, antioxidant, anti-inflammatory, antidiabetic, anti-Alzheimer’s disease, and anti-angiogenic activities of Taxifolin [29,30,31,32]. Unlike cancer cell lines, TAX has been found to reduce apoptosis in normal cells caused by inflammation, proteasome dysfunction, lipid peroxidation, and drug toxicity. In a recent experimental study, conducted by Chen et al., it was observed that exposure to DEHP induced apoptosis in chicken cardiomyocytes. However, TAX was able to counteract the toxicity of DEHP on cardiomyocytes by attenuating apoptosis [33].The low solubility and difficulty in absorption and digestion of this medication limit its bioavailability and effectiveness. To overcome these limitations, various nanoparticles, such as metal nanoparticles, liposomes, and inorganic nanoparticles, have been developed as tumor-targeting carriers for targeted drug delivery [34,35,36,37].

**Scheme 1:**
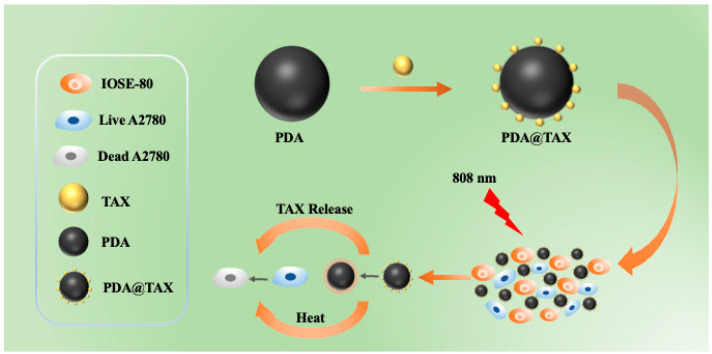
The scheme for the PDA@TAX construction route and Ovarian cancer chemo–photothermal synergistic medical therapy.

In this study, we have successfully created secure multifunctional nanoplatforms by oxidizing dopamine to polydopamine in an alkaline environment. These nanoplatforms were used to treat tumors by loading TAX (Scheme 1). We first evaluated the pH-thermal dual stimulus responsive release properties of chemotherapy–photothermal therapy and examined the effect of different polydopamine particle sizes on the loading and release of TAX. Furthermore, we demonstrated the photothermal stability of these nanoparticles as photothermal agents, with a 28.6% photothermal conversion efficiency. Our research highlights the potential of using PDA nanoparticles as a versatile loading platform for various therapeutic and photothermal agents, thanks to their molecular structure resembling melanin-like biomaterials. This study uncovers the multiple applications of PDA NPs as loading platforms for a wide range of therapeutic and photothermal agents, which can be utilized in cancer treatment. These agents have a similar molecular structure to melanin-like biomaterials, enhancing the bioavailability of TAX.

## 2. Results and Discussion

### 2.1. Preparation and Characterization

**Figure 1:**
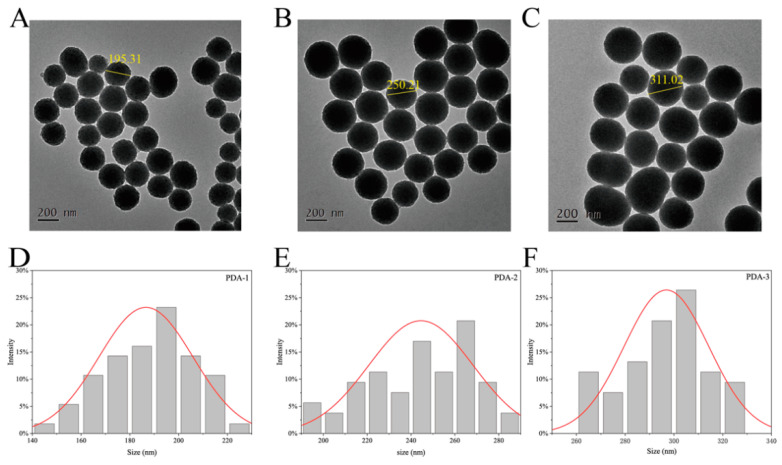
TEM micrographs of (**A**) PDA-1, (**B**) PDA-2, and (**C**) PDA-3. (**D**–**F**) Correspond to the size distribution of (**A**–**C**).

The particle diameters of TEM characterization findings are presented in Figure 1. The obtained PDAs (PDA−1, PDA−2, and PDA−3) exhibit uniform size and have a spherical shape, as depicted in Figure 1A–C. The red fitting curve in the figure shows that the average particle size is 86.6 ± 19.1 nm, 244.5 ± 18.2 nm, and 291.4 ± 22.9 nm, respectively, indicating a homogeneous distribution of nanometer sizes (Figure 1D–F). The results demonstrate that by adjusting the ammonia concentration, one can control the size of PDA NPs. Table 1 provides the hydrated particle sizes in nanometers, which are measured as 200.22 ± 1.29 nm, 280.83 ± 2.07 nm, and 328.78 ± 2.19 nm. It is important to note that the particle sizer measurement includes the water and kinetic diameter of the nanoparticles, incorporating both the nanoparticle diameter and the thickness of the hydration layer. Consequently, the particle size measured with the particle sizer appears larger than that observed in the TEM image. In order to achieve a synergistic chemotherapy–photothermal treatment effect, we utilize the surface of PDA to load TAX, which allows us to harness the photothermal effectiveness of clinical chemotherapeutic medicines. The strong molecular affinity between TAX and PDA enables the adsorption of TAX onto the surface of nanoparticles.

### 2.2. TAX Loading and Releasing

**Figure 2:**
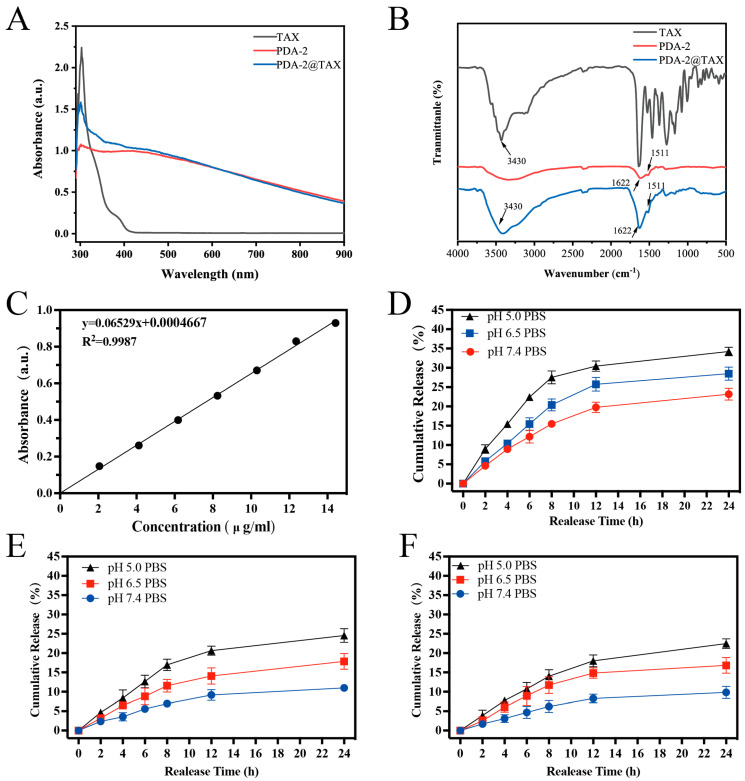
(**A**) UV-vis spectra of PDA, TAX, PDA–2@TAX. (**B**) FITR spectra of PDA, TAX, PDA–2@TAX. (**C**) standard curve of TAX. (**D**–**F**) Release profiles of TAX from PDA−1@TAX, PDA–2@TAX and PDA–3@TAX.

**Figure 3:**
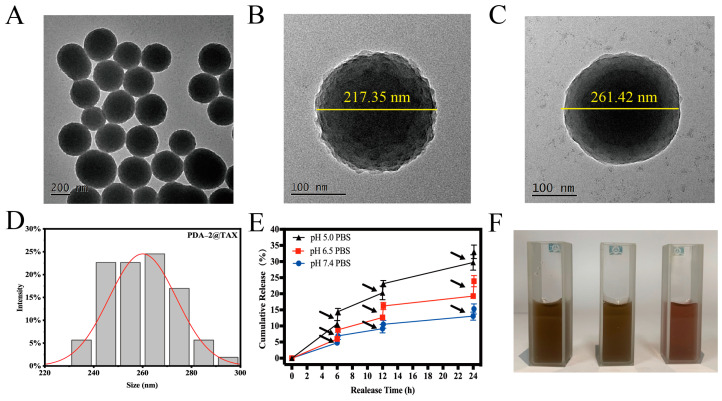
(**A**) TEM micrograph of PDA–2@TAX. (**B**,**C**) TEM micrographs of individual PDA−2 and PDA–2@TAX (**D**) Size distribution corresponding to (**A**). (**E**) Release profiles of TAX from PDA–2@TAX at pH 5.0, 6.5, 7.4, with 808 nm laser irradiation (1.5 W/cm^2^) at predetermined time points indicated by arrows. (**F**) Digital images of PDA@TAX were redispersed in different media after 24 h.

We investigated the drug loading capacity of the nanocarriers using PDA−2, which was stirred overnight in the dark until saturation TAX loading was reached. Figure 2A demonstrates that TAX was effectively incorporated into PDA−2 for drug delivery and treatment. TAX has a distinctive peak at 298 nm, and the characteristic peak of PDA−2@TAX has an increased trend from 298 nm to the same trend of absorption as PDA−2 in the 400–900 nm region. The distinctive peaks of PDA at 1511 cm^−1^ and 1622 cm^−1^ in Figure 2B are PDA−2 and PDA–2@TAX, which are linked to the weaker bending of C–N on indole-5,6-quinone and the stretching vibration peak of C–C on the indole aromatic ring. In the IR spectra, the sample of TAX displayed the TAX characteristic peak at 3430 cm^−1^, which is linked to the O–H stretching vibration in flavonoids [38]. When TAX was modified in PDA, the above characteristic peaks of PDA−2 and TAX appeared simultaneously and their characteristic peaks narrowed at 3430 cm^−1^, indicating the successful loading of TAX [39]. In Figure 3F, the proposed nanoparticles exhibited excellent stability in different solutions, including deionized water, PBS, and cell culture medium containing serum. Table 1 illustrates that the absolute value of zeta potential progressively increases with increasing particle size, indicating that the particle reaches a certain stability after expanding in size. The unloaded TAX was removed by centrifugation, and then the TAX content in the supernatant was measured using UV at 298 nm to calculate the drug loading and encapsulation rate. The standard curve of TAX is shown in Figure 2C, which demonstrates the concentration of TAX has a linear relationship in the range of 2–14 μg/mL. The loading and encapsulation rates of PDA for its three particle sizes are shown in the form, with loading rates of 20.9 ± 1.29%, 36.17 ± 2.07%, 44.79 ± 2.19%, and encapsulation rates of 6.09 ± 1.06%, 12.05 ± 0.79%, 14.93 ± 1.67%, respectively.

We used three types of PDA (PDA−1, PDA−2, and PDA−3) that were stirred overnight in the dark until saturation TAX loading was achieved. The drug loading capability of the nanocarriers was evaluated. To determine the drug loading and encapsulation rate, the unloaded TAX was removed by centrifugation. The concentration of TAX in the supernatant was determined using UV at 298 nm. Figure 2C shows the TAX standard curve, which demonstrates a linear relationship between the concentration of TAX and 214 μg/mL. Table 2 presents the loading and encapsulation rates of PDA for its three particle sizes. The loading rates were 20.9 ± 1.29%, 36.17 ± 2.07%, and 44.79 ± 2.19%, respectively, while the encapsulation rates were 6.09 ± 1.06%, 12.05 ± 0.79%, and 14.93 ± 1.67%. It was observed that as the nanoparticle size increased, the specific surface area of the particles also increased, resulting in higher encapsulation and loading rates of the medication. Subsequently, the release behavior of the PDA@TAX medication in PBS was examined at three different solution pH levels (5.0, 6.5, and 7.4). The results indicated that drug release increased with decreasing pH for all three particle sizes, suggesting that acidic conditions promote drug release in tumors (Figure 2D–F). After 24 h, the cumulative release rates for the three particle sizes in PBS at pH 5.0, which is considered the physiological environment of most cancer cells, were 33.45 ± 2.31%, 25.82 ± 1.54%, and 23.34 ± 1.96%, respectively. Moreover, drug release decreased with increased particle size. Based on the particle size stability, drug loading rate, and drug release rate, PDA-2 was selected for the next experiment.

The form of the black spheres on PDA−2 after TAX loading is shown in Figure 3A, with a slightly larger particle size. Figure 3D’s particle size distribution plot confirms this, displaying a value of 252.2 ± 13.6 nm. Table 1 demonstrates that PDA-2’s hydrated particle size and zeta potential increased upon TAX loading, indicating improved stability following drug loading. Figure 3B,C show that the PDA surface becomes smooth after loading TAX, which is attributed to TAX occupying the surface pore channel. In addition to responding to pH stimulation, this nanocarrier is also influenced by NIR light. Figure 3E shows the release of TAX from PDA@TAX at all three pH levels when exposed to the 808 nm laser. Under irradiation at pH 5.0, the PDA@TAX release increased to 8%, compared to the non-irradiated group. The pH-dependent and NIR-responsive drug release behaviors can be utilized to control drug release and enhance the anticancer cell effects.

### 2.3. In Vitro Studies of Photothermal Therapy

**Figure 4:**
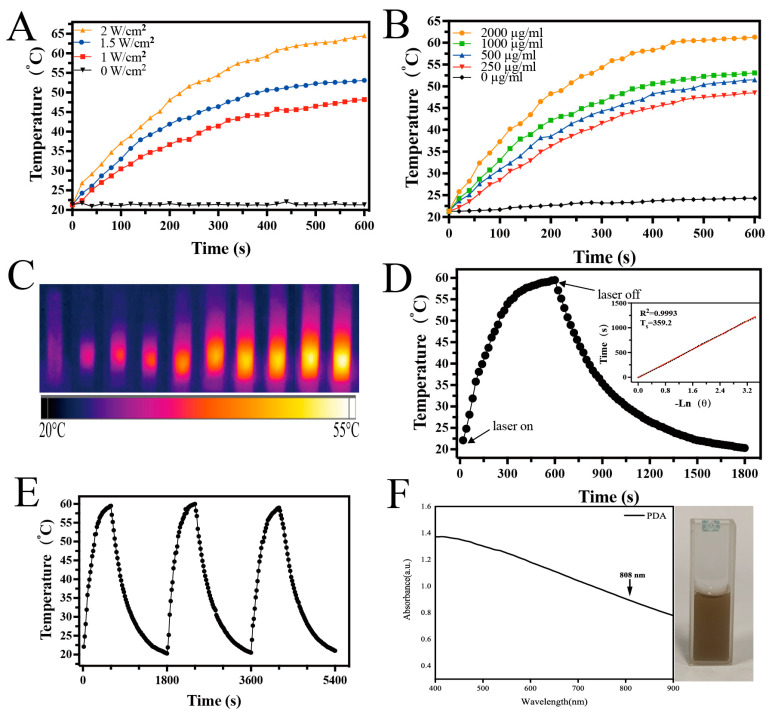
(**A**) Temperature variation of PDA−2@TAX (1000 µg/mL) under 808 nm laser irradiation with different laser densities (0, 1, 1.5, 2 W/cm^2^) for 10 min. (**B**) Temperature elevation of water and PDA−2@TAX aqueous dispersions of different concentrations (250, 500, 1000, and 2000 µg/mL) under 808 nm laser irradiation with a power density of 1.5 W/cm^2^ for 10 min. (**C**) Infrared thermoimaging of cells after irradiation for different minutes. (**D**) Photothermal response of PDA–2@TAX aqueous dispersion (1000 µg/mL, 1 mL) under NIR irradiation (808 nm, 1.5 W/cm^2^). The laser was shut off until the temperature reached a steady-state value. The inset illustrates linear time data versus −lnθ obtained from the cooling period. (**E**) The temperature change of PDA–2@TAX aqueous dispersions (1000 µg/mL, 1 mL) under 808 nm NIR light irradiation for three cycles, with 10 min irradiation followed by a 20 min cooling period. (**F**) Absorbance curve of PDA−2 aqueous solution; The photograph of the aqueous solution of PDA–2 NPs is on the right.

The PDA–2@TAX solution exhibits exceptional photothermal performance, efficiently converting light energy into heat energy when exposed to an 808 nm laser ray. Figure 4A demonstrates that it continuously increases in temperature at a given concentration of PDA–2@TAX (1000 µg/mL), and that the temperature change is positively correlated with the laser power. At a concentration of 1000 µg/mL, the nanoparticles can raise the temperature to approximately 55 °C during 10 min of light exposure, as shown in Figure 4B, with the temperature increasing with increasing nanoparticle concentration. In contrast, in the same irradiation settings, the temperature of the water remains relatively stable. The cooling and heating curve (in Figure 4D) and the UV absorption at 808 nm (in Figure 4F) is used to compute the photothermal conversion efficiency: the τ_s_ is 359.2 s, obtained by inset of 4D; hS is calculated to be 11.692 mW/°C; and the value of T_max_–T_surr_ is 37.4 °C. So the photothermal conversion efficiency (η) of PDA–2@TAX is calculated to be 28.6%, greater than (the previously observed) photothermal conversion efficiency for Polypyrroleb and PEGylated copper nanoplatforms [40,41]. According to Figure 4E, PDA–2@TAX remained unchanged during all light cycles after repeated switching of NIR irradiation, and there was no significant decrease in the maximum heating temperature. The temperature of the nanoparticle solution was monitored using an infrared thermal imaging camera over a period of ten minutes. Figure 4C shows that at an intensity of 1.5 W/cm^2^, a concentration of 1000 µg/mL of PDA–2@TAX can reach a temperature above 55 °C. These experimental findings demonstrate that PDA–2@TAX is an effective photothermal reagent due to its excellent stability.

### 2.4. In Vitro Cytotoxicity Assay

**Figure 5:**
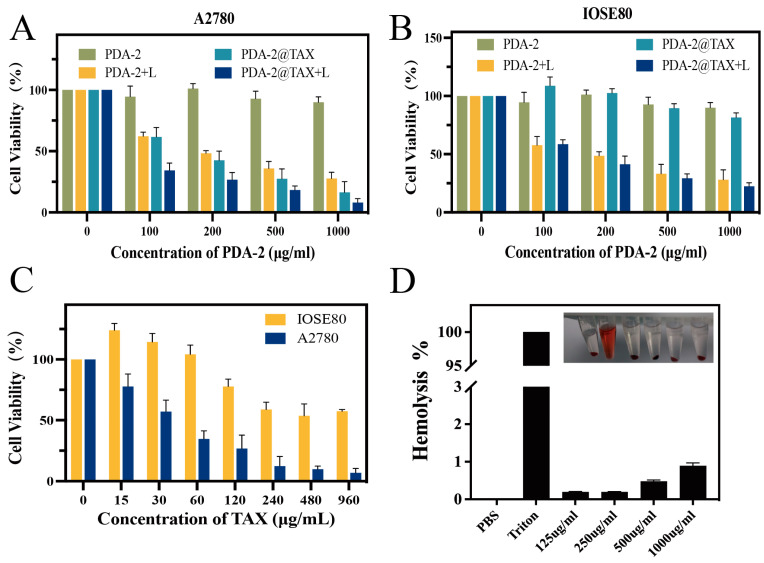
(**A**) In vitro, cell viabilities of A2780 cells incubated with various concentrations of PDA−2 and PDA–2@TAX, with or without 808 nm NIR laser irradiation (808 nm, 1.5 W/cm^2^, 6 min). (**B**) In vitro, cell viabilities of IOSE80 cells incubated with various concentrations of PDA−2 and PDA–2@TAX, with or without 808 nm NIR laser irradiation (808 nm, 1.5 W/cm^2^, 6 min). (**C**) Cell viability of A2780 cells and IOSE80 cells incubated with various concentrations of free TAX. The error bars mean standard deviations. (**D**) The hemolysis percentage with different concentrations of PDA–2 NPs (125, 250, 300, 500, and 1000 µg/mL). Triton is employed as the positive control, whilst PBS is used as the negative control. The inset illustrates representative images of hemolysis.

Human ovarian cancer A2780 cells and normal ovarian cells IOSE80 cells were utilized as experimental subjects. The intracellular temperature of cells irradiated with varying concentrations of PDA−2 and PDA−2@TAX at a power density of 1.5 W/cm^2^ (808 nm) for a duration of 6 min was measured. Subsequently, the activity of A2780 and IOSE80 cells was assessed using MTT assay, considering different concentrations of light exposure and absence of light. PDA−2 in the non-light group was evaluated after incubation with both cells, as depicted in Figure 5A, B. Even at the maximum concentration of 1000 μg/mL, the cellular activity of PDA−2 remained above 80%, indicating its good biocompatibility. However, in the PDA−2@TAX group, the cell viability decreased with increasing nanoparticle concentration after incubation with A2780. This decrease was attributed to the concentration-dependent inhibition of cell proliferation caused by the release of TAX in the acidic tumor environment. In the study, PDA–2@TAX demonstrated cell activity exceeding 80% within the 0 to 1000 μg/mL range when incubated with IOSE80 cells. At a concentration of 100 μg/mL, the nanomaterials of PDA–2@TAX displayed a beneficial impact on normal ovarian cells, which can be attributed to the anti-inflammatory and antioxidant properties of TAX [42,43,44,45]. In addition, small concentrations of TAX can enhance the proliferative effect of IOSE80. To further investigate this, we examined the activity of A2780 cells and IOSE80 cells using the TAX chemotherapeutic drug group alone. As shown in Figure 5C, the cellular activity was found to be less than 80% when the TAX concentration was 15 μg/mL. Moreover, the cell survival rate significantly decreased with an increase in TAX concentration. At a TAX concentration of 960 μg/mL, the survival rate of ovarian cancer cells decreased to 6.82%, indicating the potent tumor-killing effect of the chemotherapeutic drug group alone. However, it is worth noting that TAX had a different effect on non-cancerous IOSE80 cells. After incubation with IOSE80 cells at concentrations ranging from 15 to 60 μg/mL, TAX enhanced the cell activity. The cell activity only declined to less than 80% when TAX concentrations exceeded 120 μg/mL.

The MTT method was utilized to determine the relative cell survival of A2780 and IOSE80 when exposed to PDA–2@TAX in the light irradiation group. In this group, the ovarian cancer cells were subjected to irradiation with various concentrations of nanoparticles. Subsequently, the cells were exposed to 1.5 W/cm^2^ 808 nm near-infrared laser irradiation for 6 min, and the cell survival rate was measured. According to Figure 5A,B, the light group exhibited a higher inhibition of cellular value-added when compared to the non-light group in the same conditions. Furthermore, there was a close concentration link between the two cytotoxicities. When PDA–2@TAX was incubated with A2780, the cell survival rate was 7.99% in the light group, whereas it decreased to 16.38% in the non-light group. This outcome can be attributed to the photothermal effect of the material leading to increased TAX release, and the synergistic photothermal-chemotherapeutic effect of the material after 808 nm NIR irradiation. The results showed that when PDA–2 and PDA–2@TAX were incubated with IOSE80, there was no significant difference in cell viability between the light group and the control group, which suggests that the decrease in cell viability was primarily caused by photothermolysis. Therefore, it can be concluded that the concentration of photothermite had a significant impact on the viability of A2780 cells.

### 2.5. In Vitro Hemolysis Test

Figure 5C illustrates the outcomes. It shows that the hemolysis rate, which was measured when red blood cells were incubated with different doses of PDA−2, was below 5%. These results indicate that the substance exhibits satisfactory hemocompatibility and meets the criteria for the hemolysis test in medical materials.

### 2.6. The LIVE/DEAD Assay

**Figure 6:**
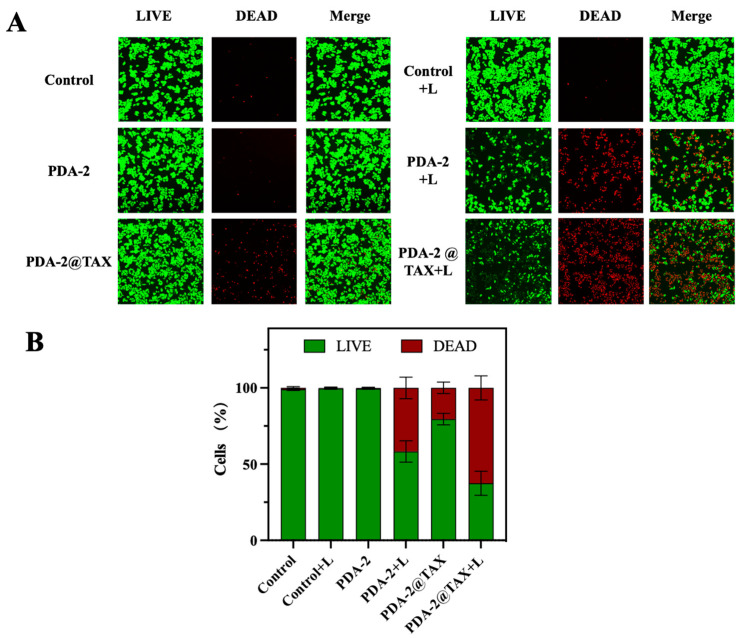
(**A**) Results of LIVE/DEAD experiment. (**B**) Quantization plots of LIVE/DEAD staining.

The LIVE/DEAD staining test results are displayed in Figure 6A. The cells were stained with PI and Calcein AM, respectively. Red fluorescent signal chemicals were produced when PI interacted with the nucleic acid of dead cells, while green fluorescent substances were produced when calcein AM reacted with active cells. The fact that the Control and Control + L blank groups in the figure are essentially green shows that the cells are essentially viable. Because the material’s photothermal action inhibited the development of A2780 cells, the live cells in the PDA−2 group remained essentially green, whereas the cells in the PDA−2 + L group displayed a minor amount of mortality. The PDA–2@TAX + L group exhibited a larger red area and a higher number of cell deaths, indicating a stronger inhibitory impact of chemotherapy and light on the proliferation and growth of A2780 cells. Figure 6B quantified the image and displayed a pattern that was consistent with cytotoxicity.

## 3. Experimental Section

### 3.1. Chemicals and Materials

Dopamine hydrochloride was purchased from Macklin Biochemical Technology Co., Ltd. (Shanghai, China). Taxifolin with a purity of 98.0% was obtained from the National Institutes for Food and Drug Control of China, batch number 111816-201102. Aqueous ammonia was purchased from Xilong Chemical Co., Ltd. (Beijing, China). Ethanol anhydrous was purchased from Xinbote Chemical Co., Ltd. (Tianjin, China). The TAX used in the experiment is from China Food and Drug Administration. A2780 cell line (human ovarian cancer cell) and IOSE80 (Human ovarian cells) were purchased from the Fuheng Biotechnology Co., Ltd. (Zhengzhou, China). Distilled water was utilized in the experiment. All chemical reagents were of analytical grade.

### 3.2. Synthesis of Polydopamine with Different Particle Size

PDA preparation followdc the previously discussed methodology [38,46]. To generate polydopamine nanoparticles of PDA–1, 22.5 mL of ultrapure water was added to a 100 mL round bottom flask. The water bath was then heated to 30 °C with magnetic stirring before 10 mL of anhydrous ethanol and 0.75 mL of NH_4_OH were successively added, and stirring was continued for 30 min. After precisely measuring and dissolving 0.125 g of dopamine hydrochloride in 2.5 mL of ultrapure water, the solution was rapidly added to the mixture and stirred continuously for 24 h. The produced polydopamine nanoparticles (PDA NPs) were centrifuged (three times at 9000 rpm) and cleaned, before being kept at room temperature. Other conditions were held constant and the quantity of NH_4_OH was decreased to 0.5 mL and 0.25 mL, respectively, for the synthesis of PDA NPs of PDA–2 and PDA–3.

### 3.3. TAX Loading and Releasing

First, 3 mL of anhydrous ethanol was used to dissolve 60,000 µg of TAX. Next, 30,000 µg of dried PDA nanoparticles from PDA−1 were weighed and added to the solution created in the previous step. The solution was centrifuged with an anhydrous ethanol solution to remove the loaded TAX after being stirred in the dark for a whole night, which resulted in a colorless supernatant. The supernatants were pooled and fixed to a volume of 100 mL, and UV-vis-NIR spectroscopy was used to determine the loading capacity of TAX. Conditions for PDA-loaded TAX were maintained the same for PDA−2 and PDA−3. The investigation of drug release at pH 5.0, 6.5, and 7.4 was followed by the collection of free TAX released from the dialysate at various time intervals. UV-Vis-NIR measurements of the TAX concentration emitted by PDA@TAX were also made.

By replicating the NIR environment with 808 nm laser irradiation, the release of TAX was encouraged. In this experiment, TAX-loaded PDA was dissolved in PBS at various pH levels (5.0, 6.5, and 7.4), and the samples were exposed to 1.5 w/cm^2^ 808 nm laser radiation for 6 min at various intervals. The solutions were collected and centrifuged to obtain the released TAX for measurement and determination by UV-Vis-NIR spectroscopy.

The drug Encapsulation Efficiency (EE) and Loading Capacity (LC) are calculated by the following equations:

(1) $$EE\left(\%\right)=\frac{W_{1}}{W_{total}}\times 100\%$$

(2) $$LC\left(\%\right)=\frac{W_{1}}{W_{2}}\times 100\%$$

The formula is as follows: W_1_ is the loaded TAX mass, W_total_ is the total TAX weight, and W_2_ is the total carrier mass.

### 3.4. Photothermal Effect of the NPs

A glass test tube was filled with 1 mL of various concentrations of aqueous PDA–2@TAX (0, 250,500, 1000, and 2000 µg/mL), and each sample was exposed to radiation for 10 min at the same power (1.5 w/cm^2^), or for 10 min at various powers (0, 1, 1.5, and 2 w/cm^2^). An electronic thermocouple thermometer was used to record the temperature at 20 s intervals. This experiment was thought to be heated for 10 min under the influence of an 808 nm laser with a power density of 1.5 W/cm^2^ and then switched off, allowing the solution to cool naturally. This was done to study the thermal stability performance of PDA–2@TAX. The heating and cooling cycles were repeated three times, and the temperature was recorded every 20 s with a thermocouple instrument.

We approximate the photothermal conversion efficiency of PDA–2@TAX based on the ramp-up and ramp-down curves of the nanoparticles at 1000 µg/mL of light for 10 min to better assess the photothermal performance of the nanoparticles. The photothermal conversion efficiency was computed as follows, according to the literature:

(3) $$\eta =\frac{\left[hS\;\left(T_{max}-T_{surr}\right)-Q_{Dis}\right]}{\left[I\left(1-10^{-A_{808}}\right)\right]}\times 100\%$$

(4) $$t=\tau_{s}\times \left(-ln\theta \right)$$

(5) $$hS=\frac{\left({Ʃ}_{i}\;m_{i}C_{p,i}\right)}{\tau_{s}}$$

(6) $$\theta =\frac{T-T_{surr}}{T_{max}-T_{surr}}$$

Heat transfer coefficient (h), exposure to radiation surface area (S), equilibrium temperature (T_max_), and ambient temperature (T_surr_) are all included in the equation. A_808_ is the absorbance of PDA–2@TAX NPs at 808 nm, Q_Dis_ is the heat of light absorbed by the glass cuvette itself, I is the laser power density, m is the mass of the sample, C_p_ is the thermal capacity of the sample, t is cooling time after irradiation, and τs is the sample system time constant.

### 3.5. In Vitro Studies on Photothermal Therapy

To investigate PDA–2@TAX’s in vitro biotoxicity and if it is appropriate for clinical use. A2780 cells and IOSE80 cells were plated in 96-well dishes at a density of 2 × 10^4^ cells/well and then incubated at 37 °C in a sterile incubator with 5% CO_2_ in 1640 medium, which contained 10% FBS and 100 U/mL penicillin-streptomycin. After 24 h of cell apposition, cells were washed three times with 100 μL of PBS. Then, 100 μL of fresh media containing various concentrations of PDA–2@TAX (0, 100, 200, 1000 μg/mL) were added to each well, and the culture was maintained for another 4 h. The cells were treated with nanoparticles for 4 h in the light-irradiated group. Similarly, we examined phototoxicity. An 808 nm laser was used to irradiate the cells for 6 min at a power density of 1.5 W/cm^2^. Following this, 10 μL of MTT (5000 µg/mL) was added to each well, and incubation was carried out for a further 4 h. In order to determine the relative vitality of the cells, the supernatant was eventually removed, 100 μL of DMSO was added to each well, and the absorbance of each well at 490 nm was measured using an enzyme marker. Additionally, the same procedure mentioned above was used to analyze the toxicity of TAX alone on cells.

### 3.6. The LIVE/DEAD Trial

24-well plates were seeded with A2780 cells in the logarithmic growth phase, and the cells were cultivated for 24 h at 37 °C in an incubator with 5% CO_2_. Following the growth of the cells to approximately 75% in each well, the 24-well plates were taken out and given two PBS washes. A total of 400 μL was poured into each well of the 24-well plates, together with the drug medium PDA−2 and PDA–2@TAX (200 μg/mL), and the plates were then incubated for four hours in the incubator. Following 4 h of incubation in the drug media, each well in the light group was exposed to 6 min of 808 nm radiation at 1.5 w/cm^2^ before being put in an incubator for a further 4 h of incubation. After removing the culture medium using a suction device and giving each well a single PBS wash, 250 μL of the Calcein AM/PI assay working solution was applied. The wells were then incubated for 30 min at 37 °C in the dark. A fluorescent microscope was used to view the staining after the incubation time.

### 3.7. Hemolysis Test

Blood from a suitable amount of rabbits was drawn, and sodium citrate was used to prevent clotting. The erythrocytes were extracted from the serum using centrifugation (3000 rpm, 10 min), washed twice in PBS solution, and then resuspended in PBS solution to create 2% erythrocyte suspension. The ultimate nanoparticle concentrations of PDA–2@TAX were 125, 250, 500, and 1000 μg/mL after 0.4 mL of diluted erythrocyte suspension was added to 0.4 mL of PDA@TAX suspension. As a negative control (0% hemolysis) and a positive control (100% hemolysis), respectively, triton and PBS buffer were utilized. Three parallel groups of each were centrifuged after 3 h of incubation at 37 °C. Then, using equipment for enzyme standardization, the absorbance values of the supernatants at 545 nm were determined.

The hemolysis rate (*HR*) was calculated according to the following equation:

(7) $$HR\left(\%\right)=\frac{Dt-Dnc}{Dpc-Dnc}\times 100\%$$

*Dt* is the absorbance of the test sample, *Dpc,* and *Dnc* are the absorbances of the positive control and negative control, respectively.

## 4. Conclusions

The study aimed to develop high-quality PDA–2@TAX NPs with uniform size, strong biocompatibility, and controlled release capabilities. These nanoparticles demonstrated promising benefits in the challenging environment of tumors, including targeted drug delivery and high photothermal conversion efficiency. In vitro anticancer tests revealed that the combination of TAX and polydopamine photothermal therapy had a synergistic effect in suppressing the growth of cancer cells A2780. The successful administration of PDA–2@TAX NPs for combined chemotherapy–photothermal treatment of antitumor drugs provides a valuable reference for targeted drug delivery and minimally invasive chemotherapy–photothermal therapy, which can significantly reduce side effects on normal tissues. Furthermore, the photothermal action and antibacterial activity of TAX make our nanoparticles suitable for clinical applications such as wound dressings and post-operative healing of melanoma.

## Figures and Tables

**Table 1:** Hydrated particle size distributions and Zeta potentials of PDA-1, PDA-2, PDA-3, and PDA-2@TAX, respectively.

Sample	Hydrated Particle Size Distributions (nm)	Zeta Potentials
PDA-1	200.22 ± 1.29	33.34 ± 6.27
PDA-2	280.83 ± 2.07	38.84 ± 0.80
PDA-3	328.78 ± 2.19	43.06 ± 0.65
PDA-2@TAX	300.78 ± 2.19	48.62 ± 0.34

**Table 2:** PDA−1, PDA−2, and PDA−3 have different rates of drug loading and encapsulation.

Sample	LC (%)	EE (%)
PDA-1	20.9 ± 1.29	6.09 ± 1.06
PDA-2	36.17 ± 2.07	12.05 ± 0.79
PDA-3	44.79 ± 2.19	14.93 ± 1.67

## Data Availability

The data presented in this study are available on request from the corresponding author.
